# Genome Sequence of *Lelliottia* sp. Strain WAP21, Isolated from Soil in Canola Fields in Victoria, Australia

**DOI:** 10.1128/mra.01018-21

**Published:** 2022-04-14

**Authors:** Phuong Nguyen Tran, Muhammad Zarul Hanifah Md Zoqratt, Agnes Michalczyk, M. Leigh Ackland

**Affiliations:** a School of Life and Environmental Sciences, Deakin University, Melbourne Burwood Campus, Burwood, Victoria, Australia; b School of Science, Monash University Malaysia, Bandar Sunway, Selangor, Malaysia; Universidad Nacional Autónoma de México

## Abstract

Here, we describe the genome of *Lelliottia* sp. strain WAP21, which was isolated from the soil of canola fields in Australia. The genome has a size of 4.9 Mbp and 4,583 predicted genes, with some potential pathways for metabolism of various carbon sources and metal acquisition.

## ANNOUNCEMENT

The genus *Lelliottia* is a relatively newly characterized genus within the *Enterobacteriaceae* family that was reclassified from Enterobacter as a novel genus based on multilocus sequence analysis (MLSA) in 2013. *Lelliottia* bacteria are Gram-negative, rod-shaped, facultative anaerobes that have been identified in diverse environments and food (1). Lelliottia amnigena was associated with onion bulb decay (2), while Lelliottia jeotgali sp. nov. was found in fermented clam (3) and Lelliottia aquatilis sp. nov. was identified in a drinking water storage reservoir (4).

*Lelliottia* sp. strain WAP21 was isolated from a rhizosphere, bulk soil, and canola root sample obtained in Bungalally, Victoria, Australia (coordinates: −36.814969, 142.307717). A suspension was made from the sample with sterile phosphate-buffered saline (PBS), and colonies were obtained by 1,000-fold dilution, spread plating on tryptone-yeast (TY) agar, and aerobic incubation at 22°C for 48 h. Single colonies confirmed with Gram stain were further streak plated on tryptic soy agar (TSA) and incubated aerobically at 30°C for 24 h. Genomic DNA was then extracted with the Monarch genomic DNA purification kit (New England Biolabs). A library was prepared from the extracted DNA with the Nextera Flex library preparation kit and sequenced on a MiSeq platform using the MiSeq reagent kit v2 (300 cycles) (Illumina, San Diego, CA, USA). A total of 1,259,356 paired-end reads with an average length of 150 bp were retained after quality and adapter trimming using Trimmomatic v0.39 (5). Default settings were used for all tools unless otherwise specified. Trimmed reads were *de novo* assembled by SPAdes v3.15.3 with a kmer size of 127 (6).

The genome, with 53 contigs (7), a G+C content of 53.5%, and a total length of 4,940,396 bp (*N*_50_, 443,208 bp), was assessed using the QUAST Web interface (http://cab.cc.spbu.ru/quast; 8); 4,583 genes were predicted with Prodigal v2.6.3 (9) and annotated with PGAP v5.3 (10).

Classification was performed by GTDB-Tk v1.5.0 against the Genome Taxonomy Database (GTDB) release 202 (11). Strain WAP21 was assigned to the *Lelliottia* genus but failed to be assigned to any existing species, with closest placement average nucleotide identity (ANI) of 93.07% to L. amnigena ZB04 (GenBank assembly accession number GCA_001652505.2) and ANIm of 84.94% to L. amnigena LMG 2784^T^ (GenBank assembly accession number GCA_002553545.1) (cutoff value, 95%).

The predicted genes were analyzed with KofamKOALA v2022-03-01 (KEGG release 101.0), and pathway analysis was conducted with an E value setting of 0.01. This analysis showed the presence of diverse carbohydrate metabolism (12). The genome possesses a complete gene module for dissimilatory nitrate reduction (DNRA), suggesting a role for *Lelliottia* sp. strain WAP21 in the soil N cycle. One previous study demonstrated that high labile C availability shifts NO_3_ consumption from denitrification to DNRA (13, 14).

### Data availability.

The nucleotide sequence of *Lelliottia* sp. strain WAP21 was submitted to GenBank under accession number JAIWPG000000000 (version JAIWPG000000000.2). The raw reads were deposited in the NCBI Sequence Read Archive (SRA) with accession number SRR16094769.
